# Acute and Chronic Exposure to Linagliptin, a Selective Inhibitor of Dipeptidyl Peptidase-4 (DPP-4), Has an Effect on Dopamine, Serotonin and Noradrenaline Level in the Striatum and Hippocampus of Rats

**DOI:** 10.3390/ijms25053008

**Published:** 2024-03-05

**Authors:** Małgorzata Łupina, Agnieszka Wąsik, Irena Baranowska-Bosiacka, Maciej Tarnowski, Tymoteusz Słowik, Piotr Listos, Jolanta Kotlińska, Danuta Kosik-Bogacka, Izabela Gutowska, Joanna Listos

**Affiliations:** 1 Department of Experimental and Clinical Pharmacology, Medical University of Lublin, Jaczewskiego 8b St., 20-090 Lublin, Poland; 2 Department of Neurochemistry, Maj Institute of Pharmacology PAS, Smetna St. 12, 31-343 Kraków, Poland; wasik@if-pan.krakow.pl; 3 Department of Biochemistry and Medical Chemistry, Pomeranian Medical University, Powstańców Wlkp. 72 Av., 70-111 Szczecin, Poland; irena.bosiacka@pum.edu.pl; 4 Department of Physiology in Health Sciences, Pomeranian Medical University, Żołnierska 54, 70-210 Szczecin, Poland; maciej.tarnowski@pum.edu.pl; 5 Experimental Medicine Center, Medical University of Lublin, Jaczewskiego 8d St., 20-090 Lublin, Poland; tymoteusz.slowik@umlub.pl; 6 Department of Pathological Anatomy, Faculty of Veterinary Medicine, University of Life Sciences, Głęboka 30, 20-612 Lublin, Poland; piotr.listos@up.lublin.pl; 7 Department of Pharmacology and Pharmacodynamics, Medical University of Lublin, Chodźki 4a St., 20-093 Lublin, Poland; jolanta.kotlinska@umlub.pl (J.K.); joanna.listos@umlub.pl (J.L.); 8 Independent Laboratory of Pharmaceutical Botany, Department of Biology and Medical Parasitology, Pomeranian Medical University in Szczecin, Powstańców Wlkp. 72, 70-111 Szczecin, Poland; danuta.kosik-bogacka@pum.edu.pl; 9 Department of Biochemistry and Human Nutrition, Pomeranian Medical University, Broniewskiego 24, 71-460 Szczecin, Poland; izabela.gutowska@pum.edu.pl

**Keywords:** linagliptin, glucagon-like peptide-1, dipeptidyl peptidase-4 inhibitor, dopamine, serotonin, noradrenaline

## Abstract

Linagliptin is a selective dipeptidyl peptidase-4 (DPP-4) inhibitor that indirectly elevates the glucagon-like peptide-1 (GLP-1) level. The aim of the present study was to check whether linagliptin has an influence on neurotransmission in rat brain. Rats were acutely and chronically exposed to linagliptin (10 and 20 mg/kg, intraperitoneally (i.p.)). Twenty-four hours later, the striatum and hippocampus were selected for further studies. In neurochemical experiments, using high-performance liquid chromatography with electrochemical detection (HPLC-ED), the concentrations of three major neurotransmitters—dopamine, serotonin and noradrenaline—and their metabolites were measured. The analysis of mRNA expression of dopamine (D1 and D2), serotonin (5-HT-1 and 5-HT-2) and noradrenaline (α1 and α2a) receptors was also investigated using real-time quantitative reverse transcription polymerase chain reaction (RQ-PCR) in the same brain areas. Linagliptin has the ability to influence the dopaminergic system. In the striatum, the elevation of dopamine and its metabolites was observed after repeated administration of that linagliptin, and in the hippocampus, a reduction in dopamine metabolism was demonstrated. Acute linagliptin exposure increases the serotonin level in both areas, while after chronic linagliptin administration a tendency for the mRNA expression of serotoninergic receptors (5-HT1A and 5-HT2A) to increase was observed. A single instance of exposure to linagliptin significantly modified the noradrenaline level in the striatum and intensified noradrenaline turnover in the hippocampus. The recognition of the interactions in the brain between DPP-4 inhibitors and neurotransmitters and/or receptors is a crucial step for finding novel discoveries in the pharmacology of DPP-4 inhibitors and raises hope for further applications of DPP-4 inhibitors in clinical practices.

## 1. Introduction

Glucagon-like peptide-1 (GLP-1), an incretin hormone, is released to reduce glycemia after food intake. It stimulates the receptors for the GLP-1 located in the β cells in the pancreas [1]. These receptors are coupled to G-protein, and their stimulation activates adenylate cyclase [2]. GLP-1 is rapidly degraded by the enzyme dipeptidyl peptidase-4 (DPP-4) [3,4]; therefore, the pharmacological effect of GLP-1 is extremely short (about 2–3 min). Currently, two groups of incretin drugs have been approved for therapy of type 2 diabetes [5]. These include GLP-1 peptide analogs and DPP-4 enzyme inhibitors. The most important advantage of these drugs is their antihyperglycemic effect without risk of hypoglycemia. 

GLP-1 is able to cross the blood–brain barrier [6], producing effects in the central nervous system (CNS). Moreover, the preproglucagon neurons, in which GLP-1 peptide is synthesized, occur in the nucleus of the solitary tract in the brainstem [7,8,9]. From here, GLP-1 neurons project widely throughout the brain and spinal cord [10,11,12], inducing a neuromodulatory effect [13]. The GLP-1 receptors occur in brain structures strongly associated with food intake regulation and rewarding and motivational behaviors, including the hypothalamus, ventral tegmental area (VTA), nucleus accumbens, amygdala, and others [12,13,14,15,16,17]. Many experiments have been performed to recognize the neuromodulatory activity of drugs affecting the GLP-1 level.

Firstly, it was noticed that GLP-1 analogs reduce appetite. This was observed after interventricular injections of GLP-1 in fasted rats [18] and after intraperitoneal injections with GLP-1 peptide in fasted mice [19]. In obese patients, the use of GLP-1 analog promoted insulin secretion [20]; reduced glycated hemoglobin, fasting blood glucose and body weight; and improved postprandial blood glucose control and β cell function [21]. Consequently, semaglutide was recently approved for use in the treatment of obesity. It is also known that GLP-1 is a mediator of the inflammatory response [22] as well as that it has an influence on neurotransmission [23], but detailed effects have not been fully recognized. In parallel, it was also evidenced that the administration of a GLP-1 analogue inhibited the rewarding effect of various addictive substances, i.e., amphetamine [24], cocaine [24,25], nicotine [26] and ethanol [27,28], in experimental animals. Thus, it was confirmed that GLP-1 receptors have an important role in rewarding effects of addictive drugs. Our previous experiments showed that administration of a DPP-4 inhibitor—linagliptin—reduced the rewarding effects of morphine in rats [29] and attenuated naloxone-induced morphine withdrawal in mice [30]. Our results suggest that GLP-1 peptide participates in morphine dependence while other mechanisms closely associated with DPP-4 inhibition may not be excluded. 

An increasing amount of evidence documents that DPP-4 inhibitors may be an important pharmacological tool. They have pleiotropic effects. In addition to their antihyperglycemic effect, DPP-4 inhibitors have a beneficial impact on the circulatory system. They also produce anti-inflammatory and antiapoptotic effects [31]. Literature data demonstrate the effect of DPP-4 inhibitors on the CNS. Pipatpiboon et al. [32] proved that vildagliptin, a DPP-4 inhibitor, improved neuron function, in rats. Additionally, a decrease in β-amyloid accumulation in the mouse model of Alzheimer’s disease was also demonstrated [33]. Moreover, the significant increase in GLP-1 level in the hippocampus and improvement of cognitive functions were observed after administration of the DPP-4 inhibitors saxagliptin and vilda-gliptin in rats [34,35]. The beneficial effects of DPP-4 inhibitors on cognition function were also observed in people suffering from diabetes mellitus type 2 and Alzheimer’s disease [36]. Literature data demonstrated that sitagliptin, an inhibitor of DPP-4, induced an increase in the level of dopamine in the striatum and tyrosine hydroxylase protein. Furthermore, decreased neuroinflammation and the reversal of neuronal loss in rats were observed [37]. The role of linagliptin in a rat model of Parkinson’s disease was also investigated. Results confirmed that linagliptin prevented motor deficits and reduced the level of inflammatory cytokines in these rats [38]. Thus, taking into consideration the similarity in pharmacological action of GLP-1 analogs and DPP-4 inhibitors, it can be assumed that an increase in GLP-1 peptide activity has an important role in the mechanism of action of DPP-4 inhibitors.

The involvement of rewarding effects in food intake regulation and three major neurotransmitters—dopamine, serotonin and noradrenaline— in neurodegeneration is well known. Therefore, the aim of the present study was to check whether linagliptin, a xanthine-based molecule that acts selectively and reversibly on the DPP-4 enzyme, has an influence on neurotransmission in the brain. To obtain it, rats received linagliptin intraperitioneally (i.p.) in two schemes: an acute dose (one dose in the morning) and chronic doses (once a day for 8 consecutive days in the morning). Twenty-four hours after linagliptin exposure, rats were decapitated, and the striatum and hippocampus were selected for further studies. In a neurochemical study, using high-performance liquid chromatography with electrochemical detection (HPLC-ED), the concentrations of three major neurotransmitters—dopamine, serotonin and noradrenaline—and their metabolites were measured. Dopamine is catabolized in two ways: via monoamine oxidase B (monoaminooxidase-dependent oxidative pathway) to the intraneuronal metabolite 3,4-dihydroxyphenylacetic acid (DOPAC) or via catechol-O-methyltransferase (COMT-dependent o-methylation pathway) to the extraneuronal metabolite 3-methoxytyramine (3-MT). The final metabolite of dopamine is homovanillic acid (HVA). The concentrations of all three dopamine metabolites were measured in that study. Serotonin is metabolized to 5-hydroxyindoleacetic acid (5-HIAA) via monoamine oxidase A, while noradrenaline, via COMT, is metabolized to normetanephrine (NMN). The levels of both 5-HIAA and NMN were also examined. Additionally, the mRNA expression of dopamine (D1 and D2), serotonin (5-HT1 and 5-HT2), and noradrenaline (α1 and α2a) receptors was also investigated using real-time quantitative reverse transcription polymerase chain reaction (RQ-PCR) in the same brain areas. The striatum and hippocampus were chosen in this study as fundamental areas governing the reward system and memory processing. The present study significantly extends current knowledge on the impact of DPP-4 inhibitors on dopamine, serotonin and noradrenaline transmission in the brain. It provides information on the direct effect of DPP-4 inhibitors on the levels of major neurotransmitters in the brain. It is important proof that should be considered in the assessment of potential mechanisms of action of DPP-4 inhibitors.

## 2. Results

### 2.1. Ex Vivo Neurochemical Studies—The Analysis of the Concentration of Dopamine, Serotonin and Noradrenaline and Their Metabolites Using HPLC-ED

#### 2.1.1. The Effect of Acute and Chronic Exposure to Linagliptin (10 and 20 mg/kg, i.p.) on the Concentration of Dopamine and Its Metabolites in the Striatum in Rats

One-way ANOVA revealed the significant effects of a single dose of linagliptin in the concentration of 3-MT (F_(2,14)_ = 8.742, *p* = 0.0034) and in the ratio of 3-MT/DA (F_(2,14)_ = 9.362, *p* = 0.0026). Statistically significant changes in DA (F_(2,31)_ = 9.944, *p* = 0.0005), DOPAC (F_(2,29)_ = 7.931, *p* = 0.0018), 3-MT (F_(2,31)_ = 7.193, *p* = 0.0027) and HVA (F_(2,31)_ = 7.322, *p* = 0.0025) concentrations were also documented as a result of chronic linagliptin administration to rats. There was no effect of chronic linagliptin administration on the analyzed metabolite/dopamine ratios; see Table 1.

The post hoc analysis (Tukey’s test) showed that a single dose of linagliptin (10 and 20 mg/kg) induced a significant increase in the 3-MT concentration (*p* < 0.05 and *p* < 0.01, respectively) and increase in the 3-MT/DA ratio (both doses *p* < 0.01) in the striatum of the rats. Chronic administration of linagliptin (10 and 20 mg kg) increased the concentration of DA (*p* < 0.01 and *p* < 0.001, respectively), DOPAC (both doses *p* < 0.01), 3-MT (*p* < 0.05 and *p* < 0.01, respectively) and HVA (*p* < 0.01 and *p* < 0.05, respectively) in the rat striatum; see Table 1.

#### 2.1.2. The Effect of Acute and Chronic Exposure to Linagliptin (10 and 20 mg/kg, i.p.) on the Concentration of Dopamine and Its Metabolites in the Hippocampus in Rats

One-way ANOVA showed significant differences in the DA (F_(2,31)_ = 6.051, *p* = 0.0060), DOPAC (F_(2,28)_ = 4.159, *p* = 0.0262), and 3-MT (F_(2,27)_ = 7.441, *p* = 0.0027) concentrations as well as in the DOPAC/DA (F_(2,29)_ = 7.095, *p* = 0.0031), 3-MT/DA (F_(2,24)_ = 5.619, *p* = 0.0100) and HVA/DA (F_(2,25)_ = 3.526, *p* = 0.0448) ratios in the hippocampus of tested rats as a consequence of a single dose of linagliptin; see Table 2.

The post hoc analysis (the Tukey test) revealed that only the higher single dose of linagliptin (20 mg/kg) produced an increase in the DA (*p* < 0.05) concentration and decrease in the DOPAC (*p* < 0.05) and 3-MT (*p* < 0.01) concentration in the hippocampus in rats. Only in the rats administered the higher single dose of linagliptin were the DOPAC/DA (*p* < 0.01), 3-MT/DA (*p* < 0.05) and HVA/DA (*p* < 0.05) ratios statistically significantly reduced; see Table 2.

Chronic administration of linagliptin did not cause statistically significant changes in the concentration of dopamine and its metabolites or in the analyzed metabolite/DA ratios in the hippocampus of the studied rodents; see Table 2.

#### 2.1.3. The Effect of Acute and Chronic Exposure to Linagliptin (10 and 20 mg/kg, i.p.) on the Concentration of Serotonin and Its Metabolite in the Striatum in Rats

One-way ANOVA demonstrated statistically significant effects of a single dose of linagliptin in Ser (F_(2,14_) = 21.13, *p* < 0.0001) and 5-HIAA (F_(2,33)_ = 14.73, *p* < 0.0001) concentrations and of chronic administration of linagliptin in Ser (F_(2,13)_ = 6.694, *p* = 0.0100) and 5-HIAA (F_(2,13)_ = 9.126, *p* = 0.0033) concentrations in the striatum of tested rodents; see Table 3. 

The post hoc analysis showed that the administration of a single dose of linagliptin (10 and 20 mg/kg) statistically significantly increased the Ser (*p* < 0.01 and *p* < 0.0001, respectively) and 5-HIAA (*p* < 0.05 and *p* < 0.0001) concentrations, but chronic administration of linagliptin, only at a lower dose (10 mg/kg), statistically significantly decreased the Ser (*p* < 0.05) and 5-HIAA (*p* < 0.05) concentrations in the rat striatum. No change in the 5-HIAA/Ser ratio was observed in the rat striatum due to the administration of linagliptin; see Table 3.

#### 2.1.4. The Effect of Acute and Chronic Exposure to Linagliptin (10 and 20 mg/kg, i.p.) on the Concentration of Serotonin and Its Metabolite in the Hippocampus in Rats

One-way ANOVA revealed statistically significant effects of a single dose of linagliptin in Ser (F_(2,14)_ = 5.253, *p* = 0.0199) and 5-HIAA (F_(2,14)_ = 12.47, *p* = 0.0008) concentrations and of chronic administration of linagliptin in the 5-HIAA (F_(2,29)_ = 11.17, *p* = 0.0003) concentration in the striatum of tested rodents; see Table 4. 

According to the Tukey test, administration of a single dose of linagliptin (10 and 20 mg/kg) caused a statistically significant increase in Ser (both doses *p* < 0.05) and 5-HIAA (both doses *p* < 0.01) concentrations. The post hoc analysis also showed that chronic administration of linagliptin, only at a higher dose (20 mg/kg), statistically significantly increased the 5-HIAA (*p* < 0.05) concentration and 5-HIAA/Ser (*p* < 0.05) ratio; see Table 4. 

#### 2.1.5. The Effect of Acute and Chronic Exposure to Linagliptin (10 and 20 mg/kg, i.p.) on the Concentration of Noradrenaline and Its Metabolite in the Striatum in Rats

One-way ANOVA showed statistically significant effects of a single dose of linagliptin in NA (F_(2,16)_ = 47.20, *p* < 0.0001) and NMN (F_(2,25)_ = 4.632, *p* = 0.0194) concentration in the rat striatum and in the NMN/NA (F_(2,27)_ = 8.183, *p* = 0.0017) ratio. No changes were observed in the rat striatum due to chronic administration of linagliptin; see Table 5. 

The post hoc analysis demonstrated that a single dose of linagliptin (10 and 20 mg/kg) statistically significantly increased the NA (both doses *p* < 0.0001) concentration and that a single, higher dose linagliptin (20 mg/kg) statistically significantly decreased the NMN (*p* < 0.05) concentration in the striatum of rats. According to the Tukey test, a single dose of linagliptin (10 and 20 mg/kg) statistically significantly decreased the NMN/NA (both doses *p* < 0.01) ratio; see Table 5. 

#### 2.1.6. The Effect of Acute and Chronic Exposure to Linagliptin (10 and 20 mg/kg, i.p.) on the Concentration of Noradrenaline and Its Metabolite in the Hippocampus in Rats

One-way ANOVA demonstrated statistically significant effects of a single dose of linagliptin in NMN (F_(2,13)_ = 25.06, *p* < 0.0001) concentrations and in the NMN/NA (F_(2,23)_ = 23.52, *p* < 0.0001) ratio. No changes were observed in the hippocampus of rats as a result of chronic administration of linagliptin; see Table 6. 

According to the Tukey test, administration of a single dose of linagliptin (10 and 20 mg/kg) induced a statistically significant decrease in NMN (*p* < 0.001 and *p* < 0.0001, respectively) concentration and statistically significant decrease in NMN/NA (both doses *p* < 0.0001); see Table 6.

### 2.2. The Analysis of mRNA Expression of Dopamine, Serotonin and Noradrenaline Receptors Using RQ-PCR in the Striatum and Hippocampus of Rats

#### 2.2.1. The Effect of Acute Exposure to Linagliptin (10 and 20 mg/kg, i.p.) on mRNA Expression of D1, D2, 5-HT1A, 5-HT2A, α1A and α2A Receptors in the Striatum and Hippocampus in Rats

One-way ANOVA showed that a single dose of linagliptin (10 and 20 mg/kg) induces no statistically significant changes in the mRNA expression of D1, D2, 5-HT1A, 5-HT2A, α1A and α2A receptors in the striatum and hippocampus of the tested animals; see Table 7.

#### 2.2.2. The Effect of Chronic Exposure to Linagliptin (10 and 20 mg/kg, i.p.) on mRNA Expression of D1, D2, 5-HT1A, 5-HT2A, α1A and α2A Receptors in the Striatum and the Hippocampus in Rats

**Figure 1:**
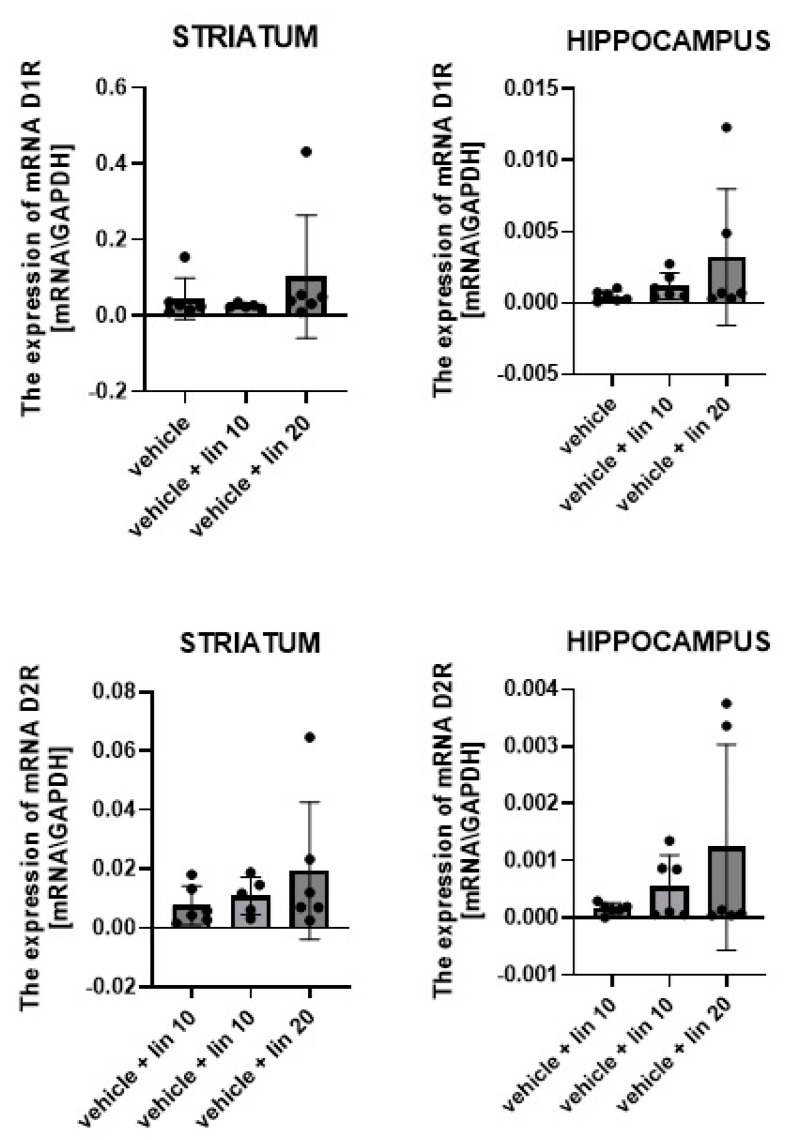
The effect of chronic exposure to linagliptin (10 and 20 mg/kg, i.p.) on mRNA expression of D1 and D2 receptors in the striatum and hippocampus in rats (n = 5–6). Data are reported as means ± SD.

One-way ANOVA showed that chronic administration of linagliptin (10 and 20 mg/kg) induces no statistically significant changes in the mRNA expression of D1 and D2 receptors in the striatum and hippocampus of the tested animals; see Figure 1.

**Figure 2:**
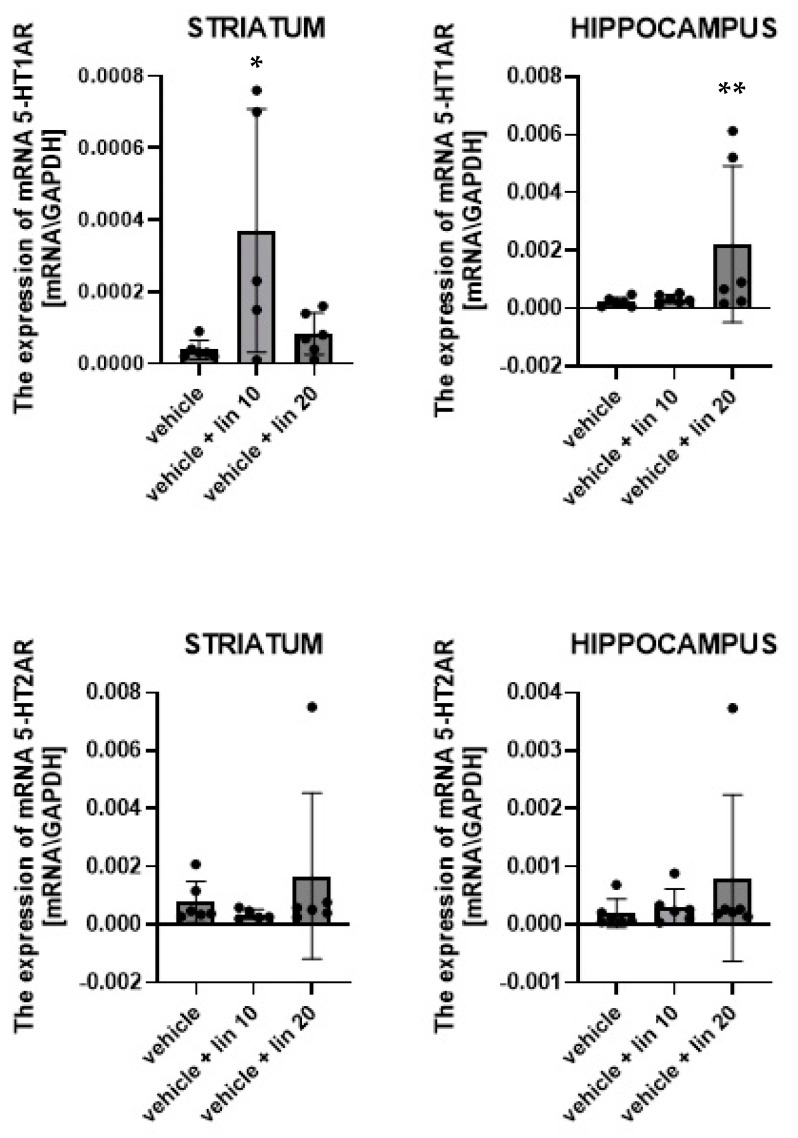
The effect of chronic exposure to linagliptin (10 and 20 mg/kg, i.p.) on mRNA expression of 5-HT1A and 5-HT2A receptors in the striatum and hippocampus in rats; * *p* < 0.05, ** *p* < 0.01 (vs. vehicle) (Tukey’s test, n = 5–6). Data are reported as means ± SD.

One-way ANOVA showed statistically significant effects of chronic administration of linagliptin in mRNA expression of 5-HT1A receptors in the striatum (F_(2,14)_ = 5.023, *p* = 0.0227) and hippocampus (F_(2,15)_ = 6.810, *p* = 0.0033) of tested rats; see Figure 2. 

The post hoc analysis demonstrated that chronic administration of linagliptin (10 and 20 mg/kg) statistically significantly increased mRNA expression of 5-HT1A receptors in the striatum (lower dose: *p* < 0.05) and hippocampus (higher dose: *p* < 0.01) in the studied rats. No more statistically significant changes were observed in the mRNA expression of 5-HT1A and 5-HT2A receptors; see Figure 2.

**Figure 3:**
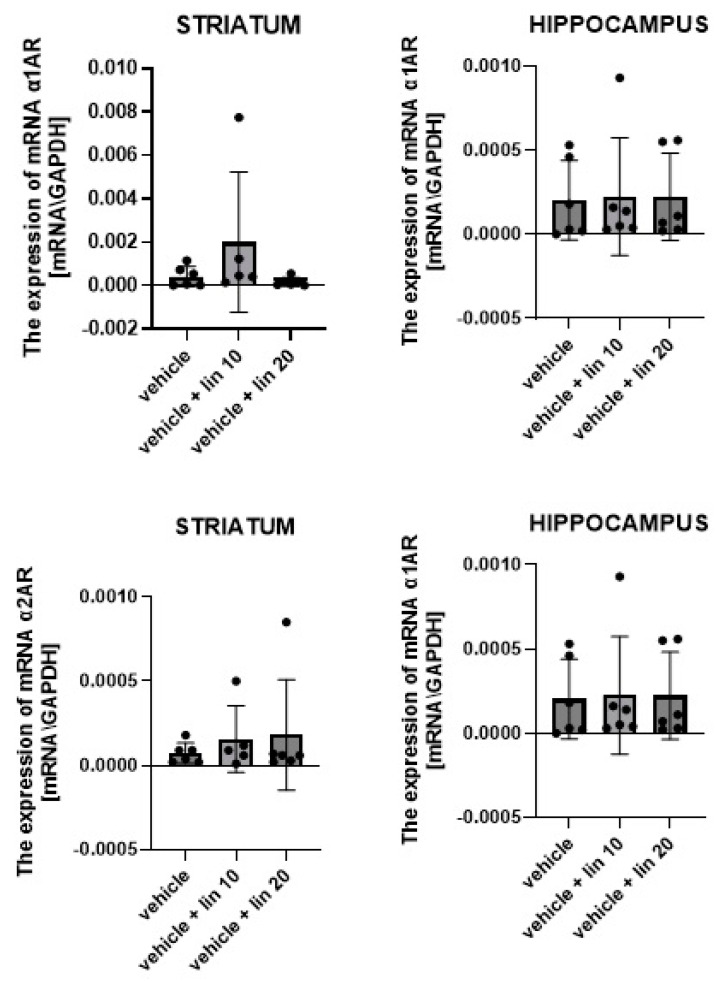
The effect of chronic exposure to linagliptin (10 and 20 mg/kg, i.p.) on mRNA expression of α1A and α2A receptors in the striatum and hippocampus in rats (n = 5–6). Data are reported as means ± SD.

One-way ANOVA showed that chronic administration of linagliptin (10 and 20 mg/kg) produces no statistically significant changes in the mRNA expression of α1A and α2A receptors in the striatum and hippocampus of the tested animals; see Figure 3.

## 3. Discussion

The balance between different neurotransmission systems in the brain is extremely important for proper functioning of the body and well-being. It is well known that three major neurotransmitters—dopamine, serotonin and noradrenaline—regulate cerebral balance. They are the basis for stabilizing mood and seeking behavior. However, it is well documented that changes in the functioning of one neurotransmitter have an impact on the activity of the other [39]. It is a result of close interconnection between these neurotransmitters. Accordingly, dopaminergic structures like the VTA, substantia nigra, nucleus accumbens, prefrontal cortex and others have an innervation from serotoninergic fibers arising from the serotoninergic raphe nuclei [40] and also receive noradrenergic projections from the nucleus tractus solitaries [41]. It is known that many parameters may modify the functioning of these major neurotransmitters and that the interpretation of obtained results may not always be clear. Therefore, in the present study, we undertook the assessment of linagliptin effects on the dopamine, serotonin and noradrenaline activity to discover its significance in the brain. However, the particular interactions between these neurotransmitters cannot be excluded. 

The major result of the reported experiments was that linagliptin, the selective DPP-4 inhibitor, influences the dopamine, serotonin, noradrenaline level in the striatum and hippocampus. Additionally, some changes in mRNA expression of selected receptors were observed. 

In the neurochemical analysis, the concentrations of dopamine, serotonin and noradrenaline and their metabolites were measured using HPLC-ED. Moreover, the intensity of metabolic changes, assessed by determining the metabolite/neurotransmitter ratio, was calculated. In experiments assessing the mRNA expression of dopamine D1 and D2, serotonin 5-HT1A and 5-HT2A and noradrenergic α1A and α2A receptors were studied using RQ-PCR. Linagliptin was given to rats in two schedules. In the first one, rats were treated with a single dose of linagliptin; in the second one, linagliptin was given chronically—once a day for 8 consecutive days.

As it was documented, linagliptin increased the level of endogenous GLP-1 peptide 2–3 times [42]. In humans, oral application of linagliptin exhibits long-term pharmacological activity (T_0_._5_ = 12 h). At present, linagliptin is approved for use in the treatment of type 2 diabetes mellitus in adults both in monotherapy or when metformin or other antidiabetics are ineffective. In the presented study, linagliptin was used in two doses (10 and 20 mg/kg), which were selected based on preliminary experiments. In that study, it was confirmed that intragastric and intraperitoneal administration of both linagliptin doses reduced glucose-induced hyperglycemia in rats but did not induce hypoglycemia (data unpublished). We decided to use intraperitoneal injections of linagliptin because we aimed for a decrease in repeated stressful situations associated with intragastric applications in rats. Intraperitoneal applications of DPP-4 inhibitors were also used by other scientists [43,44].

Moreover, the intraperitoneal administration of both doses of linagliptin inhibited the effect of morphine dependence [29,30] but did not produce any changes in the locomotor activity of mice or rats (data unpublished). Therefore, these two doses were chosen for neurochemical experiments and analysis of mRNA expression of receptors presented in that paper.

In the first step of that study, it was documented that linagliptin had an effect on the dopamine concentration both in the striatum and in the hippocampus. In the striatum, the major effect was observed after chronic administration of both linagliptin doses, and significant increases in the dopamine level and all of its metabolites were measured. In the case of a single dose of linagliptin, in the striatum, only the increases in the 3-MT concentration and 3-MT/dopamine ratio were observed. It is not clear what the reason for this was; it was possibly caused by an intensified conversion of dopamine to 3-MT or a slowdown conversion of 3-MT to HVA. It should be underlined, however, that the concentration of 3-MT is not currently considered as a reliable parameter of the activity of the dopaminergic pathway [45]. In fact, the first data on the role of 3-MT in the brain indicated its important role as an indicator of dopaminergic activity [46,47]. At present, however, it is known that in some conditions (e.g., depending on the method of animal euthanasia), 3-MT can be synthesized independently of dopamine [45]. Moreover, under certain conditions, 3-MT may act as a neuromodulator in the brain [48], playing a role as an inhibitory factor for the activity of catecholamines in the CNS [49]. It has also been documented that only about 20–30% of the amount of dopamine in the striatum is metabolized to 3-MT [50]. Therefore, in the interpretation of the results of this study, changes in 3-MT concentration were not considered as a crucial effect.

In the hippocampus, changes in the secretion of dopamine and its metabolites were also observed in the studied rats. A single dose of linagliptin significantly increased dopamine secretion and reduced dopamine metabolites. The HVA/DA ratio was reduced compared to the control group, showing a significant slowdown of dopamine metabolism in the hippocampus. In the case of the hippocampus of rats chronically treated with linagliptin, a similar tendency was observed; however, the results were not statistically significant.

In the case of acute linagliptin exposure, the changes in dopamine activity were probably too short-lived to induce receptor adaptation because, in both structures, no changes in mRNA expression of dopamine D1 and D2 receptors were observed. In the case of chronic administration of a higher dose of linagliptin, a trend towards higher mRNA expression of D1 and D2 receptors in the striatum was observed, but this effect was statistically insignificant, probably because of too high SD values. Taken together, linagliptin has the ability to influence the dopaminergic system because the elevation of dopamine and its metabolites in the striatum was observed after repeated administration of that drug, and in the hippocampus, a decrease in dopamine metabolism was demonstrated.

The interactions between GLP-1 peptide receptors and dopamine receptors have already been examined, but with varying results. Badawi et al. [51] demonstrated that both sitagliptin (DPP-4 inhibitor) and liraglutide (GLP-1 agonist) elevated the striatal dopamine level, which could be helpful in the treatment of Parkinson’s disease [51]. On the other hand, Egecioglu et al. [24], using microdialysis, demonstrated that the administration of exendin-4, a GLP-1 peptide receptor agonist, had no effect on the dopamine level in the nucleus accumbens but reduced the amphetamine- and cocaine-induced increase in dopamine release in that structure. Fortin and Roitman [52] used fast-scan cyclic voltammetry (FSCV) to study the pharmacological effects of exendin-4 on cocaine-evoked phasic dopamine signaling in the core and shell of nucleus accumbens of rats. Chronic exposure for GLP-1 receptor agonist suppressed dopamine signaling induced by cocaine in the core of nucleus accumbens but not in the shell. 

The exact mechanisms between GLP-1 receptors and the dopamine level are not fully recognized. It is assumed that stimulation of GLP-1 peptide receptors in the VTA may weaken the transmission between VTA and nucleus accumbens neurons [53] in mice. In rats, it was confirmed that the stimulation of receptors for the GLP-1 peptide in the VTA may increase dopaminergic transmission through presynaptic, glutamatergic AMPA/kainate but not N-methyl-D-aspartate (NMDA) receptor signaling [54]. It was also confirmed that activation of GLP-1 receptors in the solitary nucleus changed the expression of D2 receptors in the VTA, indicating a direct link between the solitary nucleus and the VTA via GLP-1 neurons [55]. However, given the relatively low expression of GLP-1 receptors in the VTA and nucleus accumbens [13,56], other interactions cannot be excluded. It was confirmed that the stimulation of receptors for the GLP-1 peptide could weaken the effects of addictive substances by increasing the expression of the dopamine transporter (DAT) on the surface of cells, leading to a decrease in the concentration of dopamine in the synaptic space [57]. The effect of GLP-1 peptide receptor stimulation on DAT expression has also been confirmed by other authors in in vitro studies on striatal cells [58], but not in the mouse model and not in the GLP-1 knock-out model [58]. As a growing amount of evidence suggests a correlation between diabetes mellitus and Parkinson’s disease [59], many studies have been conducted on this in animal models. Parkinson’s disease and glucose intolerance are associated with nigrostriatal dopaminergic neurons loss [60,61]. Literature data report that GLP-1 analogs improve the striatal dopamine level in patients with Parkinson’s disease and diabetes [62], protect the dopaminergic neurons in the substantia nigra and prevent dopamine loss in basal ganglia retaining control of motor functions [63,64,65]. Similar results have also been obtained using the dual GLP-1/GIP receptor agonist [66,67]. DPP-4 inhibitors also reduced symptoms of Parkinson’s disease possibly by decreasing dopamine depletion via intensification of tyrosine hydroxylase and vesicular monoamine transporter 2 (VMAT2) [51] or via stimulation of antioxidant, anti-inflammatory, antiapoptotic, neuroprotective and neurorestorative mechanisms [68,69].

In the second step of the presented study, the effect of linagliptin on serotonin was also investigated in the same brain areas. It was evidenced that acute exposure to linagliptin significantly increased the level of serotonin and its metabolites both in the striatum and in the hippocampus, but no changes in mRNA expression of serotonin receptors 5-HT1 and 5-HT2 were observed. In the case of chronic linagliptin exposure, the effect on the serotoninergic system was poorer. In the striatum, only a lower dose of linagliptin decreased serotonin and its metabolite levels and increased mRNA expression of 5-HT1 receptors. On the other hand, in the hippocampus, after a higher dose of linagliptin, an increase in serotonin metabolism and higher mRNA expression of 5-HT1 receptors were evidenced. Some modifications were also observed in the mRNA expression of 5-HT2 receptors in both structures, but these changes were statistically insignificant, probably because of too high SD values and/or relatively low expression of serotonin receptors in both areas. Moreover, the expression of other subtypes of serotonin receptor that were not the subject of these experiments may have been altered.

Some interactions between GLP-1 receptors and the serotonin system have already been described. There are reports showing the existence of interactions between GLP-1 peptide and serotonin receptors in the peripheral nervous system and in the gastrointestinal tract [70,71]. It was shown in in vitro study that the GLP-1 peptide analog (exendin-4) increased serotonin secretion in the hypothalamus in rats [72]. In another study, it was shown that chronic pharmacological blocking of 5HT2A receptors, but not 5HT2C, attenuated the anorectic effect of the GLP-1 peptide [73]. Vestlund and Jerlhag [74] showed in mice that the administration of a GLP-1 receptor analogue into the solitary nucleus increased the concentration of serotonin in the nucleus accumbens but did not affect the rate of serotonin breakdown. The obtained results suggest the existence of complex interactions in the brain between the receptors for the GLP-1 peptide and the serotoninergic system.

Thus, that study indicates that acute linagliptin exposure elevates the serotonin level in both structures. Chronic linagliptin administration produces more complex effects—a decrease in serotonin level in the striatum and an increase in serotonin turnover in the hippocampus. Moreover, a tendency towards increased mRNA expression of serotoninergic receptors 5-HT1A and 5-HT2A after chronic linagliptin treatment was observed.

It is known that stimulation of GLP-1 receptors increases the activity of the hypothalamic–pituitary–adrenal axis [75] and that GLP-1 receptors, similarly to noradrenaline receptors, are present in the solitary tract nucleus [75] in the brain. Therefore, in the last part of the presented study, the changes in the noradrenergic system were also assessed. It was documented that a single dose of linagliptin significantly increased the striatal noradrenaline level and reduced its metabolism. In the hippocampus, the effect of a single dose of linagliptin was weaker—a decrease in noradrenaline metabolism was observed. In both structures, the chronic administration of linagliptin had no effect on the concentration of noradrenaline and its rate of metabolism. Although some modifications in mRNA expression of noradrenergic receptors α1A and α2A in the striatum and hippocampus were noticed after acute and chronic linagliptin administration, these changes were statistically insignificant. The lack of statistically significant changes was probably associated with too low expression of these receptors in both structures and too high SD values. 

Taken together, although GLP-1 neurons do not colocalize with catecholamine neurons [76], in the presented experiments, the effect of acute linagliptin exposure on noradrenaline activity in the striatum and hippocampus was clearly observed. It should be emphasized that the current knowledge on the interactions between GLP-1 receptors and the noradrenaline system are poorly described, and further research in this area is necessary. Thus, the present study is the first to document the existing interactions between linagliptin and the noradrenaline system. 

A weak point of this study is the lack of protein level analysis. Undoubtedly, it would significantly extend a view on the influence of linagliptin on the changes in the brain, at the molecular level. Unfortunately, during the performance of the experiments, there was no financial possibility to realize this.

Summing up, our study provides evidence for the existence of interactions in the brain. The peripheral administration of an acute dose of linagliptin elevates both the serotonin and noradrenaline levels, and chronic linagliptin exposure has an influence on the dopaminergic system. All of these neurotransmitters are strongly involved in mental disorders, including depression, schizophrenia, abuse disorder, and others. Currently, drugs affecting DPP-4 inhibitors have been approved for use in the treatment of type 2 diabetes mellitus. Conducting further experiments is necessary to recognize their full significance in behavior such as depressive effects, anxiety and schizophrenia. The recognition of the interactions in the brain between GLP-1 drugs and neurotransmitters and/or receptors is a crucial step for finding novel discoveries in pharmacology of GLP-1 drugs. Our results raise hope for further applications of GLP-1 drugs in clinical practices.

## 4. Materials and Methods

### 4.1. Animals

The experiments were performed on male Wistar rats (160–200 g). The animals were fed a standard pelleted diet of Murigran (Agropol, Motycz, Poland) and provided with water ad libitum. During the experiments, six to eight animals were kept per cage at room temperature (22 ± 1 °C) and exposed to a normal day/night cycle. All of the experiment procedures were carried out between 8:00 a.m. and 11:00 a.m. The study was performed according to the National Institute of Health Guidelines for the Care and Use of Laboratory Animals and the European Community Council Directive for Care and Use of Laboratory Animals and was approved (14/2017) by the Local Ethics Committee (The Medical University of Lublin Committee on the Use and Care of Animals).

### 4.2. Drug

Linagliptin (MedChem Express, Monmouth Junction, NJ, USA)—a selective DPP-4 inhibitor—was used in the experiments. Linagliptin was first suspended in 5 drops (about 0.095 mL) of ethanol 96° and then subsequently diluted in 100 mL of warm (80 °C) 0.9% saline. The final concentration of ethanol was below 0.1% (precisely 0.095%). The dose of linagliptin stock solution was 25 mg/kg. To obtain linagliptin solution at doses of 10 mg/kg and 20 mg/kg, the linagliptin stock solution was diluted with 0.9% saline in ratios of 1.5:1 and 4:1, respectively. The solution for the vehicle group was prepared analogically and was given at respective timepoints. All the substances were delivered i.p. in volumes of 5 mL/kg. Linagliptin was injected at the dose of 10 and 20 mg/kg, i.p.

### 4.3. Experimental Procedure

To assess the effects of short- and long-term administration of linagliptin on the neurotransmitter levels (dopamine, serotonin and noradrenaline) and their metabolites and on the expression of mRNA receptors (dopamine receptors—D1, D2; serotonin receptors—5-HT1A and 5-HT2A; and noradrenaline receptors—α1A and α2A) in the striatum and hippocampus of rats, linagliptin was administered in two schedules. In the first, rats were treated with a single dose of linagliptin. In the second, rats received 1 dose of linagliptin per day for 8 consecutive days (8 injections in total). In both schedules, 24 h after the last linagliptin exposure, the rats were decapitated and their brain structures (the striatum and hippocampus) were dissected. Control animals received injections of the vehicle. 

**Scheme 1:**
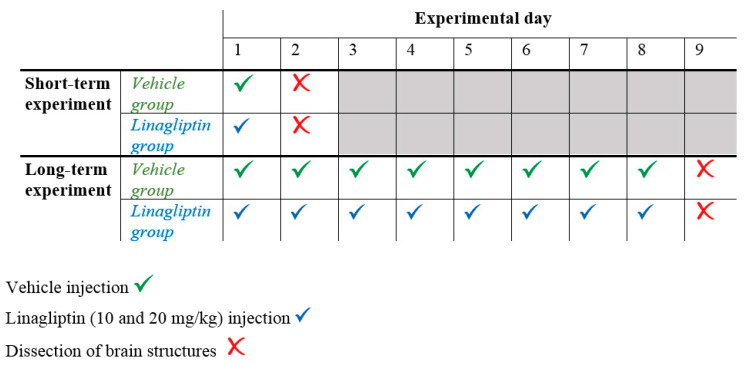
Schedule of linagliptin administration.

The experimental protocol is graphically depicted in Scheme 1.

### 4.4. Neurochemical Analysis—Ex Vivo Biochemical Studies Assessed Using HPLC-ED in the Striatum and Hippocampus of Rats

Twenty-four hours after the last vehicle/linagliptin injection, the rats were killed by decapitation, and their brain structures, including the striatum and hippocampus, were immediately dissected. The obtained tissues were frozen on liquid nitrogen (−80 °C) and stored until biochemical assay. Dopamine and its metabolites, DOPAC, 3-MT, and HVA; serotonin and its metabolite, 5-HIAA; and noradrenaline and its metabolite, NMN, were assayed using HPLC-ED. An HP 1050 chromatograph (Golden, CO, USA) was equipped with C18 columns. Tissue samples were weighed and homogenized in ice-cold 0.1 M perchloroacetic acid containing 0.05 mM of ascorbic acid. After centrifugation (10,000× *g*, 5 min), the supernatants were filtered through RC 58 0.2 µm cellulose membranes (Bioanalytical Systems, West Lafayette, IN, USA). The mobile phase consisted of 0.05 M citrate-phosphate buffer, pH 3.5, 0.1 mM of EDTA, 1 mM of sodium octyl sulfonate, and 3.5% methanol. The flow rate was maintained at 1 mL/min. dopamine, Ser, NA and their metabolites were quantified by peak height comparisons with standards run on the day of analysis. The temperature of the column and the voltage of the detector were set at 30 °C and +750 mV, respectively.

### 4.5. Analysis of mRNA Expression of Dopamine, Serotonin and Noradrenaline Receptors Using RQ-PCR in the Striatum and Hippocampus of Rats

Total RNA was extracted from 50 to 100 mg brain samples using an RNeasy Lipid Tissue Mini Kit (Qiagen, Hong Kong, China) and qualitative and quantitative analysis of isolated RNA were performed using a NanoDrop ND-1000 spectrophotometer (NanoDrop Technologies, Wilmington, NC, USA). Next, cDNA was obtained from 1 μg of total cellular RNA in 20 μL of reaction volume, using a First Strand cDNA synthesis kit and oligo-dT primers (Fermentas, Waltham, MA, USA). A quantitative assessment of mRNA levels was performed using RQ-PCR on an ABI 7500Fast instrument with Power SYBR Green PCR Master Mix reagent. The real-time conditions, i.e., 95 °C (15 s), 40 cycles at 95 °C (15 s), and 60 °C (1 min), were maintained. In compliance with melting point analysis, only one PCR product was amplified under those conditions. Each sample was analyzed in two technical replicates, and the mean Ct values were used for further analysis. The relative quantity of the target, normalized to the endogenous control Gapdh gene and relative to a calibrator, is expressed as 2^−∆∆Ct^ (−fold difference), where Ct is the threshold cycle, ∆Ct = (Ct of target genes) − (Ct of endogenous control gene, β-2 microglobulin), and ∆∆Ct = (∆Ct of samples for the target gene) − (∆Ct of calibrator for the target gene). The following primer pairs were used:

D1R F: CGC GTA GAC TCT GAG ATT CTG AAT T,

D1R R: GAG TTA AGG AGC CAC CAC ATC AGT;

D2R F: TGA CAG TCC TGC CAA ACC AGA GAA,

D2R R: TGG GCA TGG TCT GGA TCT CAA AGA;

HT51AF: CGT GCA CCA TCA GCA AGG A;

HT51AR: CTG AAG ATG CGC CCG TAG AGA;

HT52AF: ACC GCT ATG TCG CCA TCC A;

HT52AR: GAC CTT CGA ATC ATC CTG TAG TCC A;

ADRA1AF: CGA ATC CAG TGT CTT CGC AG;

ADRA1AR: ACC ATG TCT CTG TGC TGT CCC;

ADRA2AF: TCC CGC CAC TCA TCT CCA TA;

ADRA2AR: CGT TAA TCT TGC AGC TCG GC;

Gapdh F: ATG ACT CTA CCC ACG GCA AG;

Gapdh R: CTG GAA GAT GGT GAT GGG TT.

The neurochemical results are presented in the table as the arithmetical mean concentration values  ±  standard error of the mean (SEM). The dopamine oxidation rate, the dopamine oxomethylation rate and the total dopamine catabolism rate were calculated using the formula ([DOPAC]/[DA)] × 100, ([3 − MT]/] [DA]) × 100, ([HVA]/[DA]) × 100, respectively. The rate of total serotonin catabolism was calculated using the formula ([5-HIAA]/[Ser]) × 100, and the rate of total noradrenaline was calculated using the formula ([NMN])/[NA]) × 100. These results were compared to a vehicle group. After confirming a normal distribution, analysis of variance (one-way ANOVA) was applied, using the Graph-Pad Prism Software package (version 8.02). Tukey’s test was applied to perform the post hoc comparisons. A probability value at *p* < 0.05 was considered as statistically significant. Every group was composed of 5–7 rats. 

The results of mRNA expression analysis are presented in the figures as the arithmetical mean value of mRNA receptor expression  ±  standard deviation (SD). After determining a normal distribution, analysis of variance (one-way ANOVA) was applied using the Graph-Pad Prism Software package (version 8.02). The post hoc comparisons were conducted using Tukey’s test. A probability value at *p* < 0.05 was considered as statistically significant. Each group consisted of 5–7 rats.

## Figures and Tables

**Table 1:** Effect of linagliptin (10 and 20 mg/kg, i.p.) on the concentration of dopamine and its metabolites in the striatum in rats; DA—dopamine, DOPAC-3,4-dihydroxyphenylacetic acid, 3-MT—3-methoxytyramine, HVA—homovanillic acid; * *p* < 0.05, ** *p* < 0.01, *** *p* < 0.001 (vs. vehicle group) (Tukey’s test, n = 5–7). Data are reported as means ± SEM.

			DA (ng/g Tissue)	DOPAC (ng/g Tissue)	3-MT (ng/g Tissue)	HVA (ng/g Tissue)	(DOPAC/DA) × 100	(3-MT/DA) × 100	(HVA/DA) × 100
Striatum	Single doe of linagliptin	vehicle	10,508.0 ± 902.1	1309.0 ± 78.74	286.6 ± 41.62	1292.0 ± 106.9	13.53 ± 0.3970	2.771 ± 0.3841	11.87 ± 0.7049
		vehicle + linagliptin 10	10,669.0 ± 504.6	1524.0 ± 94.70	525.3 ± 40.24 *	1361.0 ± 71.05	14.58 ± 0.7214	5.053 ± 0.4216 **	13.01 ± 0.3776
		vehicle + linagliptin 20	10,793.0 ± 314.6	1477.0 ± 101.9	556.5 ± 58.28 **	1353.0 ± 110.9	14.05 ± 0.5923	5.314 ± 0.4909 **	12.83 ± 0.6536
	Chronic administration of linagliptin	vehicle	10,291.0 ± 851.6	1373.0 ± 128.0	287.7 ± 28.34	1291.0 ± 78.44	13.95 ± 0.5291	3.080 ± 0.3014	12.60 ± 0.6355
		vehicle + linagliptin 10	13,392.0 ± 303.8 **	2026.0 ± 135.8 **	428.8 ± 34.18 *	1665.0 ± 43.77 **	14.38 ± 1.688	2.945 ± 0.4348	12.51 ± 0.5049
		vehicle + linagliptin 20	13,996.0 ± 560.9 ***	1971.0 ± 132.8 **	497.0 ± 47.71 **	1551.0 ± 79.22 *	13.04 ± 1.205	3.499 ± 0.3576	11.01 ± 0.3662

**Table 2:** Effect of linagliptin (10 and 20 mg/kg, i.p.) on the concentration of dopamine and its metabolites in the hippocampus in rats; DA—dopamine, DOPAC—3,4-dihydroxyphenylacetic acid, 3-MT—3-methoxytyramine, HVA—homovanillic acid; * *p* < 0.05, ** *p* < 0.01 (vs. vehicle group) (Tukey’s test, n = 5–7). Data are reported as means ± SEM.

			DA (ng/g Tissue)	DOPAC (ng/g Tissue)	3-MT (ng/g Tissue)	HVA (ng/g Tissue)	(DOPAC/DA) × 100	(3-MT/DA) × 100	(HVA/DA) × 100
Hippocampus	Single doe of linagliptin	vehicle	13.62 ± 1.726	7.020 ± 1.405	7.920 ± 1.194	13.54 ± 2.962	57.60 ± 12.57	54.99 ± 8.728	89.42 ± 26.14
		vehicle + linagliptin 10	11.90 ± 1.266	5.440 ± 0.7359	6.780 ± 1.175	7.500 ± 0.8972	46.20 ± 5.802	51.21 ± 12.92	58.79 ± 5.910
		vehicle + linagliptin 20	19.60 ± 1.770 *	3.264 ± 0.4847 *	2.800 ± 0.3266 **	6.075 ± 0.9232	18.64 ± 2.797 **	15.13 ± 0.7017 *	26.42 ± 3.261 *
	Chronic administration of linagliptin	vehicle	15.86 ± 1.689	8.500 ± 1.268	7.850 ± 1.230	14.34 ± 2.422	43.53 ± 7.601	49.24 ± 10.89	87.41 ± 28.82
		vehicle + linagliptin 10	27.67 ± 3.471	9.500 ± 1.848	6.200 ± 0.3887	18.33 ± 2.741	26.06 ± 3.601	21.52 ± 3.145	52.40 ± 6.793
		vehicle + linagliptin 20	25.67 ± 4.224	6.333 ± 1.856	9.000 ± 0.8018	13.25 ± 0.9402	30.46 ± 5.142	31.96 ± 2.943	54.07 ± 6.820

**Table 3:** Effect of linagliptin (10 and 20 mg/kg, i.p.) on the concentration of serotonin and its metabolites in the striatum in rats; Ser—serotonin, 5-HIAA—5-hydroxyindoleacetic acid; * *p* < 0.05, ** *p* < 0.01, **** *p* < 0.0001 (vs. vehicle group) (Tukey’s test, n = 5–7). Data are reported as means ± SEM.

			Ser (ng/g Tissue)	5-HIAA (ng/g Tissue)	(5-HIAA/Ser) × 100
Striatum	Single dose of linagliptin	vehicle	268.5 ± 10.92	392.9 ± 15.01	133.2 ± 2.433
		vehicle + linagliptin 10	348.8 ± 23.67 **	455.8 ± 15.91 *	133.7 ± 3.687
		vehicle + linagliptin 20	390.7 ± 4.022 ****	510.5 ± 16.74 ****	130.8 ± 4.506
	Chronic administration of linagliptin	vehicle	276.0 ± 9.716	397.5 ± 25.56	132.1 ± 2.371
		vehicle + linagliptin 10	202.4 ± 20.07 *	264.8 ± 12.39 *	145.5 ± 11.72
		vehicle + linagliptin 20	273.2 ± 15.72	467.3 ± 40.43	157.0 ± 10.60

**Table 4:** Effect of linagliptin (10 and 20 mg/kg, i.p.) on the concentration of serotonin and its metabolites in the hippocampus of rats; Ser—serotonin, 5-HIAA—5-hydroxyindoleacetic acid; * *p* < 0.05, ** *p* < 0.01 (vs. vehicle group) (Tukey’s test, n = 5–7). Data are reported as means ± SEM.

			Ser (ng/g Tissue)	5-HIAA (ng/g Tissue)	(5-HIAA/Ser) × 100
Hippocampus	Single dose of linagliptin	vehicle	165.0 ± 15.37	238.0 ± 34.56	144.6 ± 8.653
		vehicle + linagliptin 10	244.4 ± 24.64 *	388.4 ± 22.55 **	162.3 ± 6.242
		vehicle + linagliptin 20	241.0 ± 19.93 *	404.5 ± 17.91 **	172.0 ± 8.768
	Chronic administration of linagliptin	vehicle	161.4 ± 18.30	244.3 ± 27.72	154.5 ± 11.94
		vehicle + linagliptin 10	131.4 ± 13.60	150.8 ± 13.66	112.6 ± 14.79
		vehicle + linagliptin 20	153.0 ± 18.23	406.2 ± 59.31 *	232.1 ± 29.61 *

**Table 5:** Effect of linagliptin (10 and 20 mg/kg, i.p.) on the concentration of noradrenaline and its metabolites in the striatum in rats; NA—noradrenaline, NMN—methoxynoradrenaline; * *p* < 0.05, ** *p* < 0.01, **** *p* < 0.0001 (vs. vehicle group) (Tukey’s test, n = 5–7). Data are reported as means ± SEM.

			NA (ng/g Tissue)	NMN (ng/g Tissue)	(NMN/NA) × 100
Striatum	Single dose of linagliptin	vehicle	316.4 ± 14.32	5.250 ± 1.656	1.643 ± 0.5179
		vehicle + linagliptin 10	549.5 ± 24.73 ****	2.280 ± 0.2947	0.4692 ± 0.05262 **
		vehicle + linagliptin 20	613.0 ± 30.35 ****	1.760 ± 0.1893 *	0.2922 ± 0.03848 **
	Chronic administration of linagliptin	vehicle	305.8 ± 11.41	5.140 ± 1.962	1.882 ± 0.5059
		vehicle + linagliptin 10	318.3 ± 15.36	3.486 ± 1.035	1.55 ± 0.2218
		vehicle + linagliptin 20	286.3 ± 15.58	4.129 ± 0.9987	1.212 ± 0.1997

**Table 6:** Effect of linagliptin (10 and 20 mg/kg, i.p.) on the concentration of noradrenaline and its metabolites in the hippocampus in rats; NA—noradrenaline, NMN—methoxynoradrenaline; *** *p* < 0.001, **** *p* < 0.0001 (vs. vehicle group) (Tukey’s test, n = 5–6). Data are reported as means ± SEM.

			NA (ng/g Tissue)	NMN (ng/g Tissue)	(NMN/NA) × 100
Hippocampus	Single dose of linagliptin	vehicle	402.2 ± 28.55	30.00 ± 5.060	7.389 ± 2.050
		vehicle + linagliptin 10	391.3 ± 11.79	5.340 ± 1.093 ***	1.346 ± 0.2132 ****
		vehicle + linagliptin 20	380.4 ± 39.10	4.733 ± 0.8401 ****	1.352 ± 0.2107 ****
	Chronic administration of linagliptin	vehicle	425.6 ± 28.79	29.75 ± 6.524	7.062 ± 1.621
		vehicle + linagliptin 10	530.0 ± 32.50	27.60 ± 1.536	5.808 ± 0.5894
		vehicle + linagliptin 20	482.5 ± 67.61	29.00 ± 6.688	5.226 ± 0.2510

**Table 7:** The effect of acute exposure to linagliptin (10 and 20 mg/kg, i.p.) on mRNA expression of D1, D2, 5-HT1A, 5-HT2A, α1A and α2A receptors in the striatum and hippocampus in rats (n = 5–7). Data are reported as means ± SD.

	mRNA	D1 [mRNA/ GAPDH]	D2 [mRNA/ GAPDH]	5-HT1A [mRNA/ GAPDH]	5-HT2A [mRNA/ GAPDH]	α1A [mRNA/ GAPDH]	α2A [mRNA/ GAPDH]
Expression Drugs							
Striatum	vehicle	0.02542 ± 0.008658	0.007618 ± 0.006601	0.00003833 ± 0.00002639	0.000534 ± 0.0003628	0.0003375 ± 0.0003526	0.000052 ± 0.00003564
	Vehicle + linagliptin 10	0.028 ± 5.01 × 10^−3^	0.00678 ± 3.43 × 10^−3^	0.00004137 ± 0.0000357	0.000603 ± 0.000144	0.00029 ± 0.000142	0.000038 ± 2.51 × 10^−5^
	vehicle + linagliptin 20	0.021 ± 3.25 × 10^−3^	0.00812 ± 4.21 × 10^−3^	0.00006519 ± 0.0000298	0.000419 ± 0.00017	0.000381 ± 0.000201	0.000063 ± 2.89 × 10^−5^
Hippocampus	vehicle	0.0004933 ± 0.0003678	0.0002 ± 6.12 × 10^−5^	0.000228 ± 0.000163	0.0002 ± 0.000226	7.67 × 10^−5^ ± 0.00008963	0.00003 ± 2.58 × 10^−5^
	vehicle + linagliptin 10	5.42 × 10^−4^ ± 3.19 × 10^−4^	2.40 × 10^−4^ ± 3.20 × 10^−4^	0.000261 ± 1.29 × 10^−4^	0.000143 ± 7.89 × 10^−5^	6.31 × 10^−5^ ± 4.21 × 10^−5^	2.40 × 10^−5^ ± 1.42 × 10^−5^
	vehicle + linagliptin 20	4.36 × 10^−4^ ± 2.80 × 10^−4^	2.61 × 10^−4^ ± 2.36 × 10^−4^	0.000249 ± 7.89 × 10^−5^	0.000098 ± 2.94 × 10^−5^	6.99 × 10^−5^ ± 5.90 × 10^−5^	2.81 × 10^−5^ ± 1.99 × 10^−5^

## Data Availability

The datasets generated and analyzed during the current study are available from the corresponding author on request.

## Abbreviations

3-MT	3-methoxytyramine
5-HIAA	5-hydroxyindoleacetic acid
CNS	the central nervous system
COMT	catechol-O-methyltransferase
DAT	dopamine transporter
DOPAC	3,4-dihydroxyphenylacetic acid
DPP-4 enzyme	the dipeptidyl peptidase-4 enzyme
FSCV	fast-scan cyclic voltammetry
GLP-1	the glucagon-like peptide-1
HVA	homovanillic acid
i.p.	intraperitoneally
HPLC-ED	high-performance liquid chromatography with electrochemical detection
NA	noradrenaline
NMDA	N-methyl-D-aspartate
NMN	normetanephrine
RQ-PCR	real-time quantitative reverse transcription polymerase chain reaction
SD	standard deviation
SEM	standard error of the mean
Ser	serotonin
VMAT2	vesicular monoamine transporter 2
VTA	ventral tegmental area
